# Supplementary figures and images for: Highly multiplexed genome engineering using CRISPR/Cas9 gRNA arrays
Source: PLoS One. 2018 Sep 17;13(9):e0198714. doi: 10.1371/journal.pone.0198714 (PMC6141065; doi:10.1371/journal.pone.0198714)

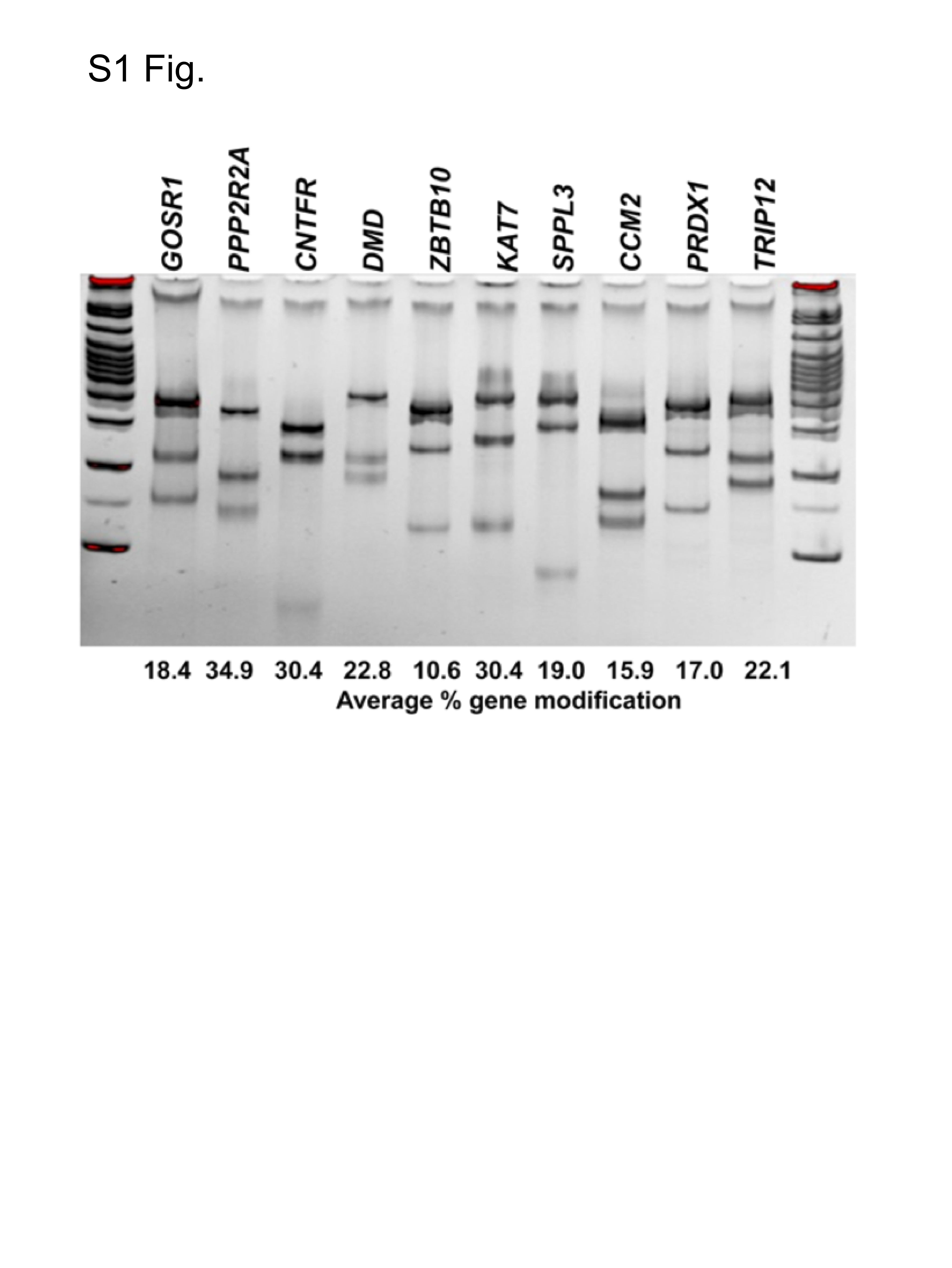

Supplement: S1 Fig — Single U6 driven gRNAs were transfected into HEK293T cells along with Cas9 encoding plasmids. Three days post transfection genomic DNA was collected and Surveyor nuclease assay performed. Mutation frequencies were assessed by densitometry of Surveyor Nuclease assay results with means of triplicate measurements shown. (TIFF) [file pone.0198714.s001.tiff]

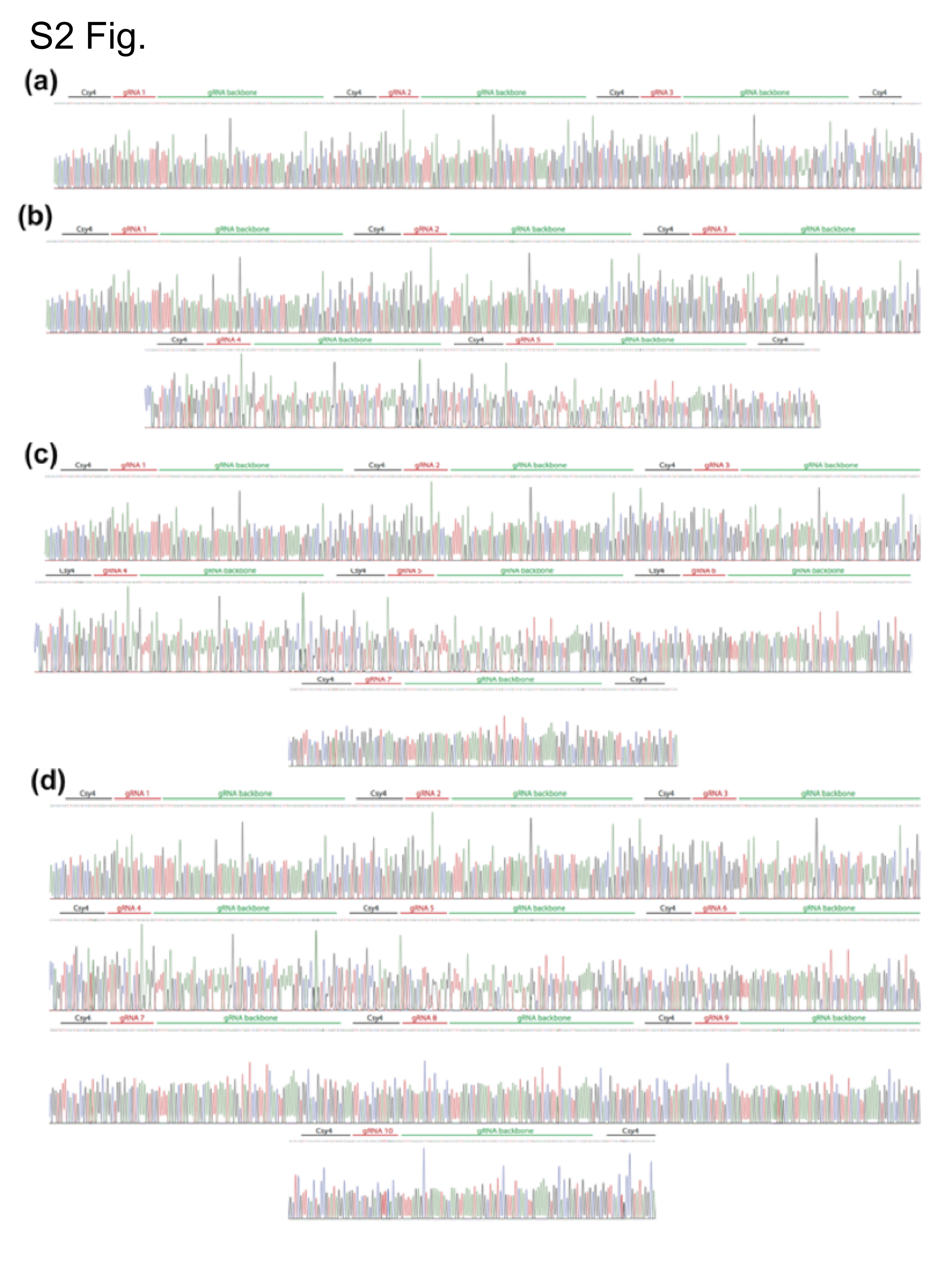

Supplement: S2 Fig — gRNA arrays containing 3 (a), 5 (b), 7 (c), or 10 (d) gRNAs. Golden gate assembled arrays were sequenced using standard Sanger sequencing using M13 forward and reverse primers. Sequences were analyzed using the Vector NTI software package (Life Technologies). (TIFF) [file pone.0198714.s002.tiff]

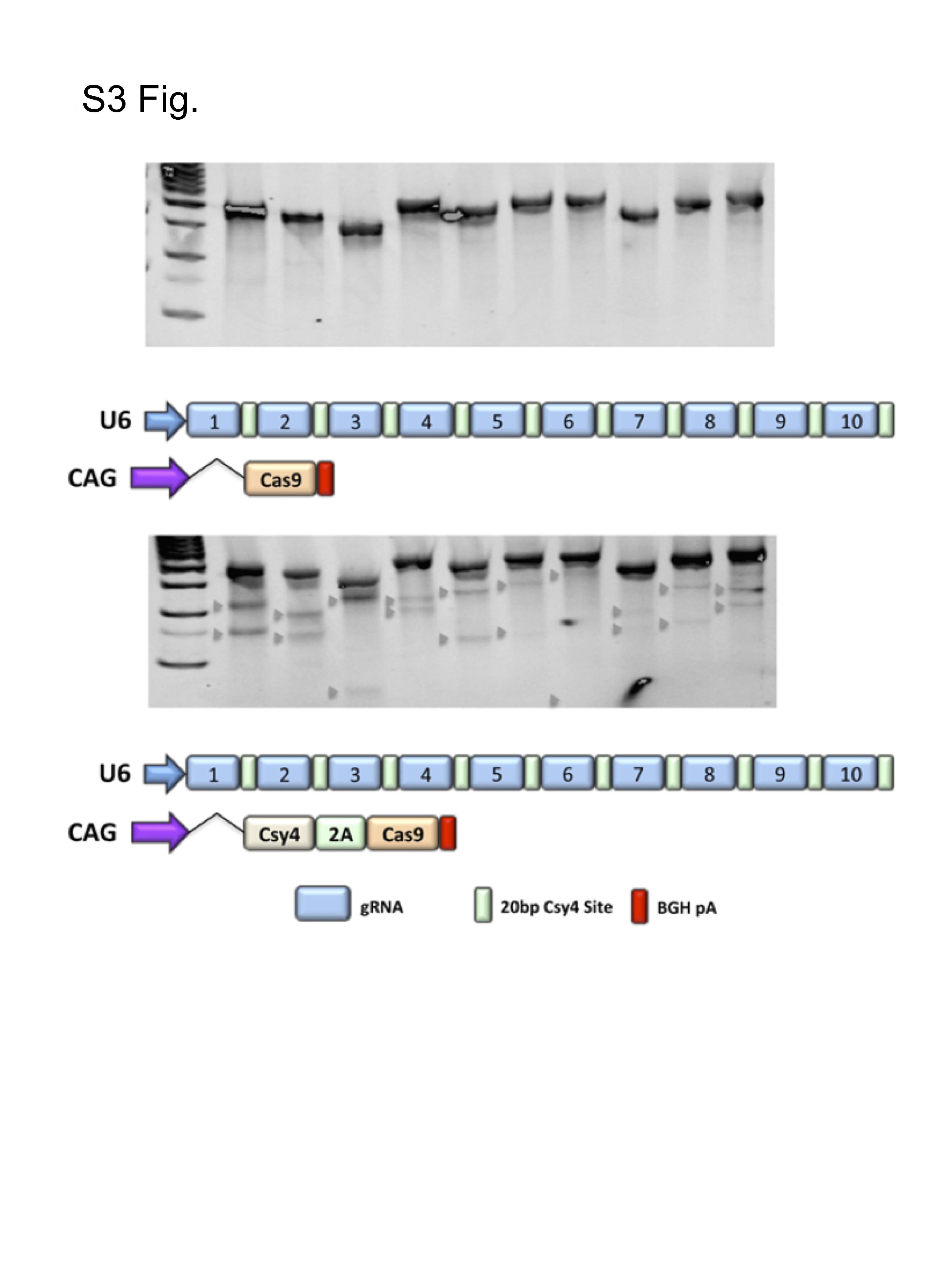

Supplement: S3 Fig — Results of surveyor nuclease assay performed on genomic DNA of HEK293T cells transfected with a 10 gRNA array and Cas9 with or without Csy4 three days post transfection. (TIFF) [file pone.0198714.s003.tiff]

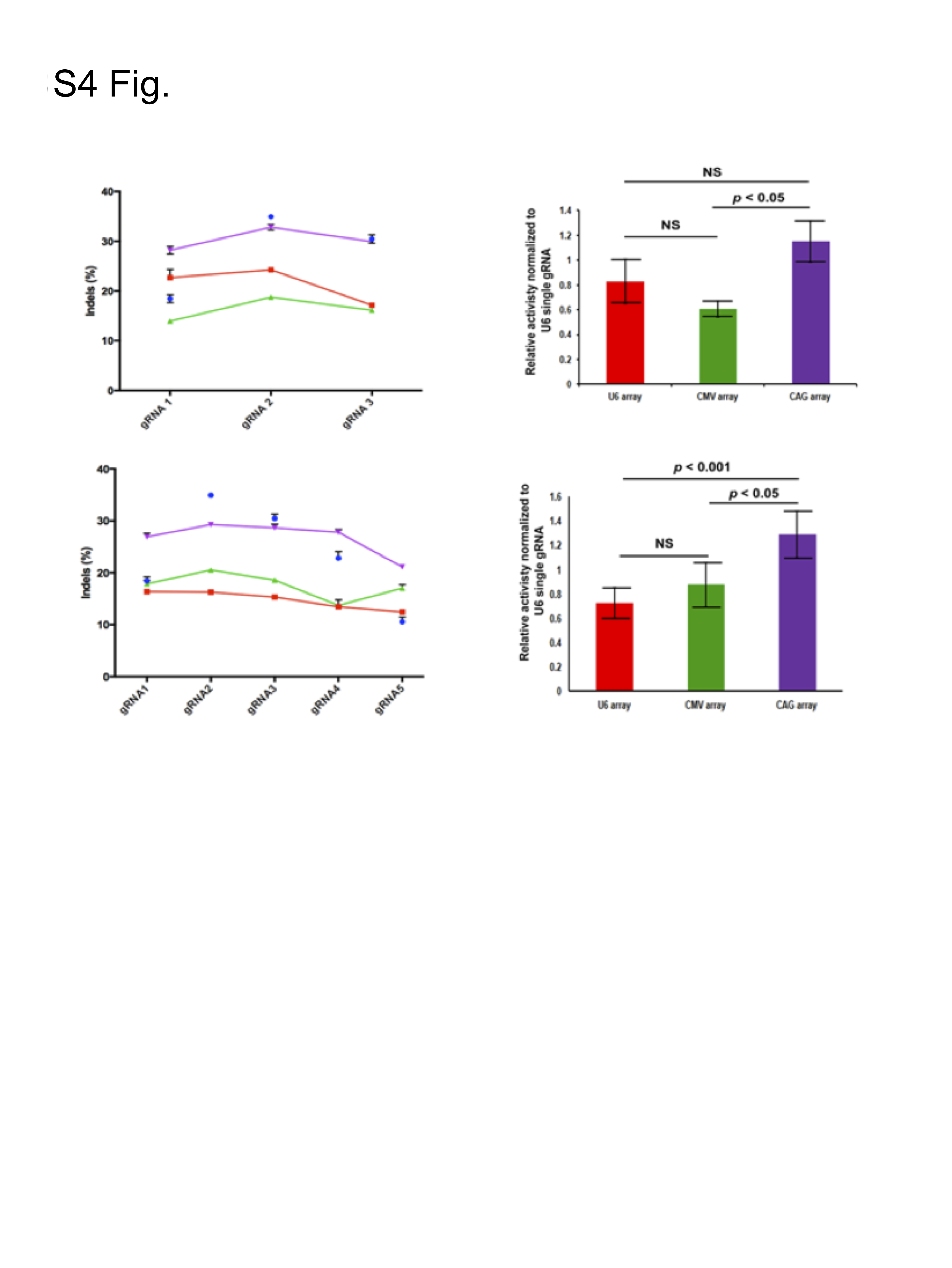

Supplement: S4 Fig — Line graphs (left) depicting the gene editing frequency of each gRNA when expressed as individual gRNAs transcribed from the standard U6 pol III promoter (blue dots) or in a single 3 (a), 5 (b) gRNA array transcribed from the standard U6 pol III promoter (red line), CMV promoter with BGH polyadenylation signal (green line), and CAG promoter with BGH polyadenylation signal (purple line) 3 days post transfection. Bar graphs (right) depicting the average gene editing frequency of the 3 (a), 5 (b) gRNA arrays expressed from each promoter normalized to the editing frequency of each individual gRNA transcribed from the standard U6 pol III promoter. Mutation frequencies were assessed by Surveyor Nuclease assay with means of triplicate measurements shown. (TIFF) [file pone.0198714.s004.tiff]

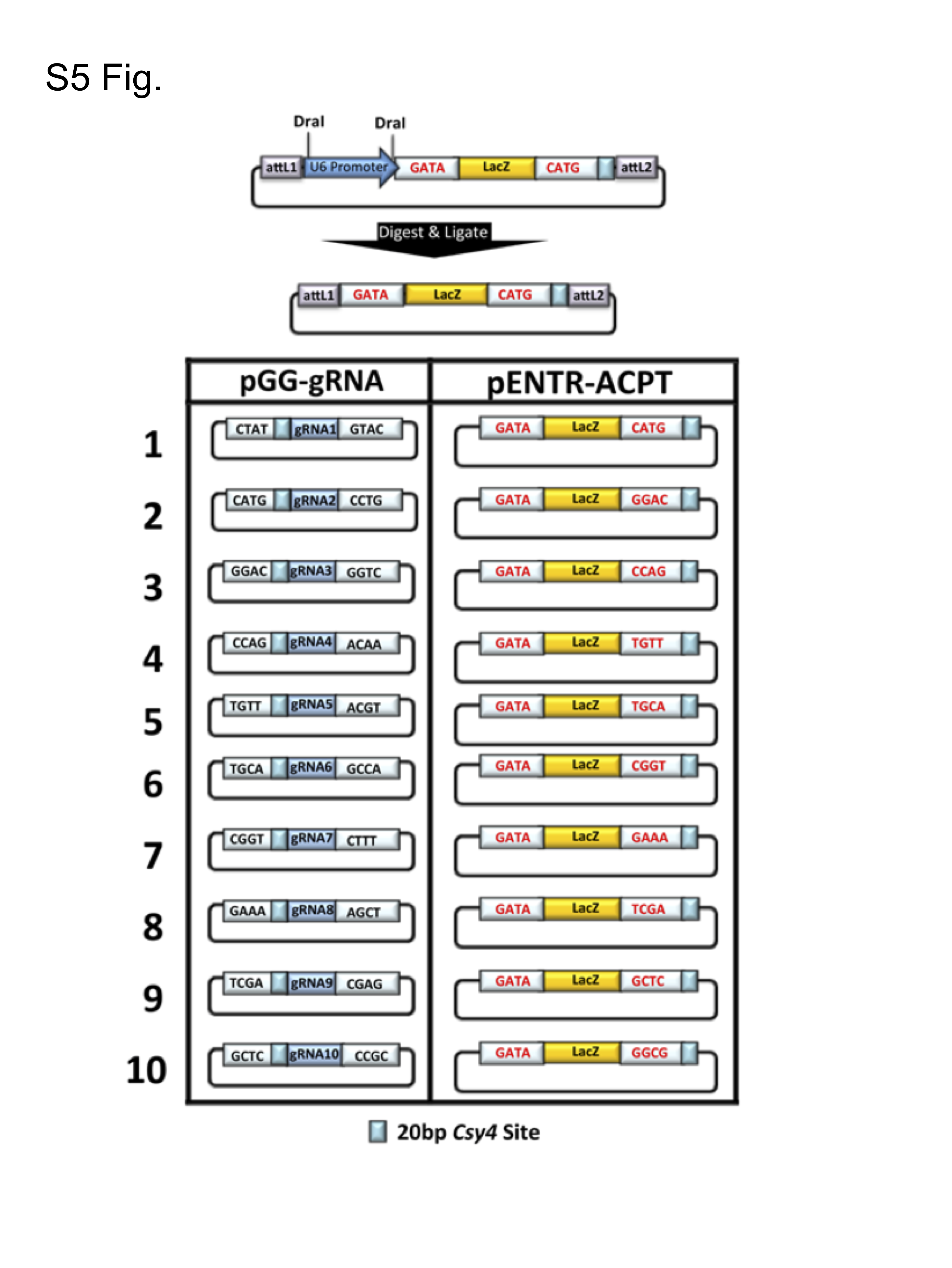

Supplement: S5 Fig — Diagram depicting the cloning strategy to remove the U6 promoter from the pENTR-ACPT 1–10 plasmids used for gRNA array assembly. Plasmids were treated with DraI (blunt) and subsequently self ligated and sequence verified. (TIFF) [file pone.0198714.s005.tiff]

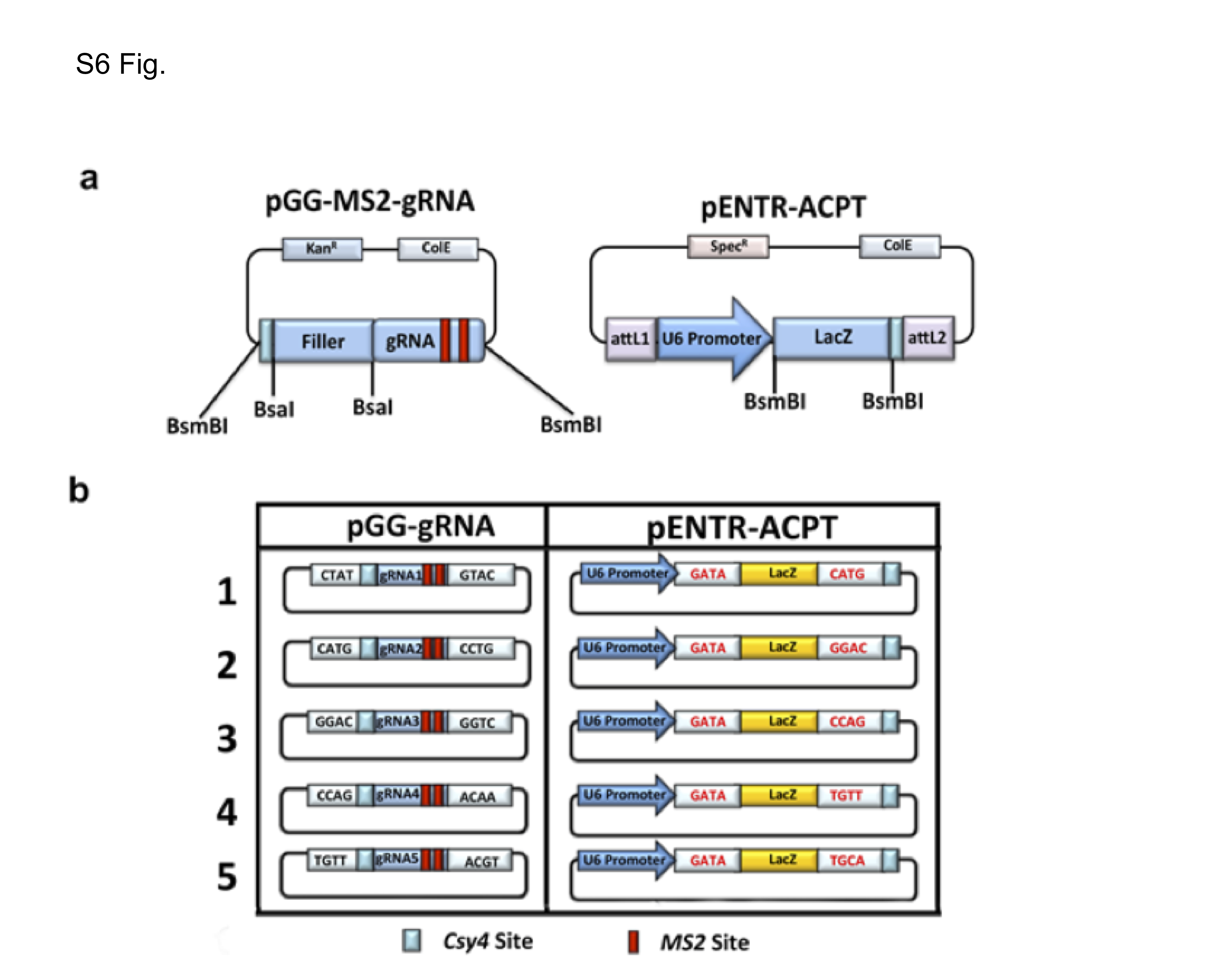

Supplement: S6 Fig — Diagram of the base pGG-MS2 (Left) and pENTR-ACPT (Right) plasmids highlighting the type IIS restriction enzymes used for protospacer oligonucleotide ligation (BsaI) and golden gate assembly (BsmBI). In addition, the pGG-MS2 cassette contains a filler sequence that is removed upon oligonucleotide ligation and a 5’ Csy4 site (light green) for array processing once assembled and expressed. A terminal Csy4 site was included in the pENTR-ACPT cassette to remove additional plasmid sequence from the terminal gRNA when expressed and a LacZ gene that is removed upon golden gate assembly to allow for blue/white colony selection. (b) Diagram of the final 5 pGG-MS2 and 5 pENTR-ACPT plasmids for assembly of arrays containing 1–5 gRNAs. The gateway attL1/2 sites of pENTR-ACPT plasmids have been left out for simplicity. (TIFF) [file pone.0198714.s006.tiff]

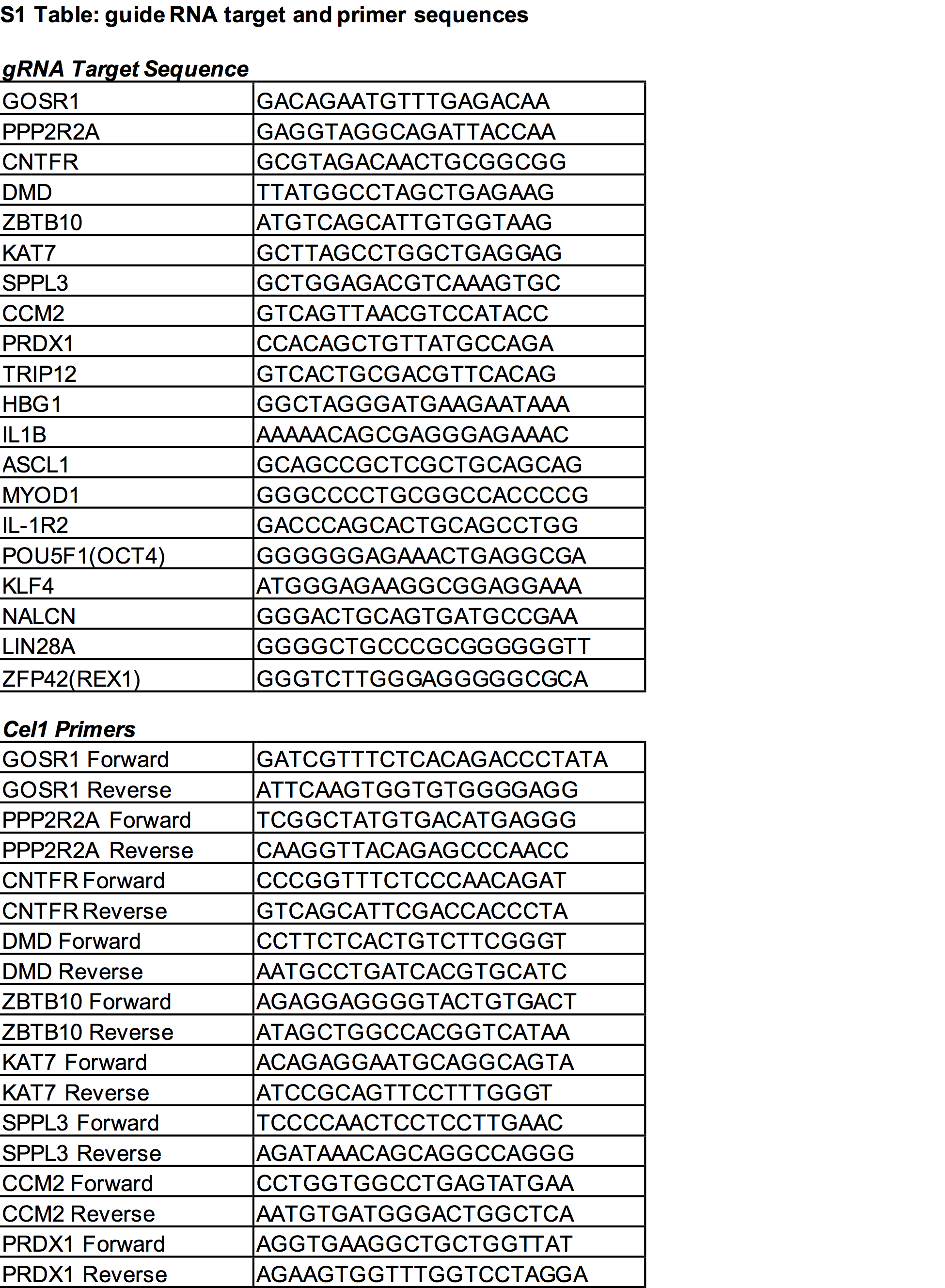

Supplement: S1 Table — List of all Cel I primers used for Surveyor nuclease analysis and oligonucleotides encoding protospacer target sequences that were cloned into pGG vectors. (TIFF) [file pone.0198714.s007.tiff]
