# Supplementary material for: Highly multiplexed genome engineering using CRISPR/Cas9 gRNA arrays
Source: PLoS One. 2018 Sep 17;13(9):e0198714. doi: 10.1371/journal.pone.0198714 (PMC6141065; doi:10.1371/journal.pone.0198714)
Supplement: S1 Protocol — (DOCX) [file pone.0198714.s008.docx]

**S1 Protocol**

**Protocol for Rapid Generation of gRNA arrays for highly multiplexed CRISPR/Cas9 gene editing and modification**

**Lab of Dr. Branden Moriarity, University of Minnesota, Department of Pediatrics**

**August 16^th^, 2018**

pGG and pACPT plasmids outlined in this paper were generated using gBlock gene fragments from IDT and are available as a library by request from authors. See supplementary materials of *Kurata et al., 2018, PLOS ONE* for ApE maps.

**Inserting unique crRNA’s into pENTR221 to create pGG 1-10 for array construction**

*To clone the guide sequence into the sgRNA scaffold, two oligos must be synthesized as shown:*

Protospacer_________

5’-GCAG**G**NNNNNNNNNNNNNNNNNNNN-3’

3’-**C**NNNNNNNNNNNNNNNNNNNNCAAA-5’

*Bold G:C is necessary for U6 promoter*

**Assembling pGG plasmids:**

1. Digest 1 ug of pENTR221 with BsmBI restriction enzyme for 1 hr at 37°C:

1 ug Plasmid

1 uL CIP

1 uL BsmBI

5 uL Tango Buffer

X uL H2O

**-------------------------------------------------------**

**50 uL Total**

1. Gel purify 2735 bp product using QIAquick Gel Extraction Kit (Qiagen) elute in TE. Store at -20°C.
2. Phosphorylate and anneal each pair of oligos:

1 uL of oligo 1 (100mM)

1 uL of oligo 2 (100mM)

1 uL of 10X T4 Ligation Buffer (NEB)

6.5 uL of ddH_2_O

0.5 uL of T4 PNK (NEB)

**-------------------------------------------------------**

**10 uL Total**

**Thermocycler protocol:**

37°C - 30 min; 95°C – 5 min; ramp -5°C/min to 25°C; 4°C – hold

(Dilute annealed oligos 1:200 for step 4)

1. Ligate backbone and oligo insert:

X uL 50 ng BsmBI digested pENTR221 plasmid from step 1

1 uL Phosphorylated and annealed oligos from step 3 (1:200 dilution)

1 uL T4 DNA Ligast Buffer (NEB)

X uL ddH2O

**-----------------------------------------------------------------------------**

10 uL Subtotal

1 uL T4 DNA Ligase (NEB)

**-----------------------------------------------------------------------------**

**11 uL Total**

1. Transform reaction into DH10β *E. coli* and plate on LB with kanamycin
2. Sequence validate with crRNA of interest inserted using M13 Fwd and M13 Rev primers

**Golden Gate Assembling arrays into pENTR - ACPT plasmids:**

Combine appropriate plasmids with appropriate pENTR - ACPT vector:

pGG1 + pGG2 combine with pENTR-ACPT2

pGG1 + pGG2 + pGG3 combine with pENTR-ACPT3

pGG1 + pGG2 + pGG3 + pGG4 combine with pENTR-ACPT4

•••

pGG[1 + 2 + 3 + 4 + 5 + 6 + 7 + 8 + 9 + 10] combine with pENTR-ACPT10

1. Golden Gate Assembly Reaction Mix:

“GGn” Assembly

150 ng pGG1

150 ng pGG2

150 ng pGG3

•••

150 ng “pGGn”*

150 ng pENTR-ACPTn

1 uL T4 DNA Ligase (NEB)

1 uL T4 DNA Ligase Buffer (NEB)

X uL ddH2O

1 uL BsaI

**-------------------------------------------------------**

**20 uL**

**Note that the 150ng of pGGn for each unique gRNA in the array is used, while only 150 ng of pENTR-ACPTn is used -- no matter the number of unique gRNAs. This is true whether “n” is 3,5, or 10 unique gRNAs.*

**Thermocycler protocol:**

5 x [37°C – 5 min; 16°C – 10 min]; 50°C – 5 min; 80°C – 5 min

Transform reaction into DH10β *E. coli* and plate on LB with spectinomycin and X-gal, select white colonies. Mini-prep and sequence validate with M13 Fwd, M13 Rev primers, and a unique oligo for mid-sequence coverage.
